# Clinical Advice by Voice Assistants on Postpartum Depression: Cross-Sectional Investigation Using Apple Siri, Amazon Alexa, Google Assistant, and Microsoft Cortana

**DOI:** 10.2196/24045

**Published:** 2021-01-11

**Authors:** Samuel Yang, Jennifer Lee, Emre Sezgin, Jeffrey Bridge, Simon Lin

**Affiliations:** 1 The Ohio State University University Wexner Medical Center Columbus, OH United States; 2 Nationwide Children's Hospital Columbus, OH United States; 3 College of Medicine The Ohio State University Columbus, OH United States; 4 Abigail Wexner Research Institute at Nationwide Children's Hospital Columbus, OH United States

**Keywords:** voice assistant, virtual assistant, conversational agent, postpartum depression, mobile health, mental health

## Abstract

**Background:**

A voice assistant (VA) is inanimate audio-interfaced software augmented with artificial intelligence, capable of 2-way dialogue, and increasingly used to access health care advice. Postpartum depression (PPD) is a common perinatal mood disorder with an annual estimated cost of $14.2 billion. Only a small percentage of PPD patients seek care due to lack of screening and insufficient knowledge of the disease, and this is, therefore, a prime candidate for a VA-based digital health intervention.

**Objective:**

In order to understand the capability of VAs, our aim was to assess VA responses to PPD questions in terms of accuracy, verbal response, and clinically appropriate advice given.

**Methods:**

This cross-sectional study examined four VAs (Apple Siri, Amazon Alexa, Google Assistant, and Microsoft Cortana) installed on two mobile devices in early 2020. We posed 14 questions to each VA that were retrieved from the American College of Obstetricians and Gynecologists (ACOG) patient-focused Frequently Asked Questions (FAQ) on PPD. We scored the VA responses according to accuracy of speech recognition, presence of a verbal response, and clinically appropriate advice in accordance with ACOG FAQ, which were assessed by two board-certified physicians.

**Results:**

Accurate recognition of the query ranged from 79% to 100%. Verbal response ranged from 36% to 79%. If no verbal response was given, queries were treated like a web search between 33% and 89% of the time. Clinically appropriate advice given by VA ranged from 14% to 29%. We compared the category proportions using the Fisher exact test. No single VA statistically outperformed other VAs in the three performance categories. Additional observations showed that two VAs (Google Assistant and Microsoft Cortana) included advertisements in their responses.

**Conclusions:**

While the best performing VA gave clinically appropriate advice to 29% of the PPD questions, all four VAs taken together achieved 64% clinically appropriate advice. All four VAs performed well in accurately recognizing a PPD query, but no VA achieved even a 30% threshold for providing clinically appropriate PPD information. Technology companies and clinical organizations should partner to improve guidance, screen patients for mental health disorders, and educate patients on potential treatment.

## Introduction

A voice assistant (VA) is inanimate audio-interfaced software augmented with artificial intelligence and capable of 2-way dialogue [1]. In 2020, 27% of all web searches used Google Assistant [2], and the adoption of VA-enabled speakers (eg, Amazon Echo) is increasing, with an estimated $3.5 billion in spending in the United States by 2021 [3]. In the last 4 years, VA as a digital health tool has been evaluated for information seeking regarding a healthy lifestyle [4], addiction [5], vaccination [6], mental health, and interpersonal violence [7]. Mental health stands out as a prime candidate for VA-based digital health intervention to fulfill technology’s promise to provide adaptive and personalized care [8,9].

Specifically, for postpartum depression (PPD), the occurrence of a depressive disorder within 12 months of delivering a baby, VA could provide timely health information. PPD is the most common obstetric complication in the United States, with an estimated 900,000 annual cases and only a small percentage of patients seeking care [10-12]. When considering reduced economic output and income loss with increased health care costs in a child of a mother with untreated perinatal mood disorder, the estimated societal cost of untreated patients may reach $14.2 billion annually in the United States [13]. PPD identification and treatment is hampered by patients’ misperceptions of the disease and benefits of treatment, along with a lack of screening and discussion with the care provider [12]. In recognition of the severity of the need, the US Preventive Services Task Force, American College of Obstetricians and Gynecologists (ACOG), and American Academy of Pediatrics all recommend regular screening for PPD and early referral for treatment [10,11,14]. In response to the screening recommendations, we evaluated VA responses to PPD questions in terms of accuracy of speech recognition, presence of a verbal response, and clinically appropriate advice given to assess the capability of VAs for digital health interventions.

## Methods

We tested four popular VAs: Amazon Alexa, Apple Siri, Google Assistant, and Microsoft Cortana [15]. Apple Siri was used on an iPhone 11 Pro (Apple Corp) while the other three VAs were installed on a Pixel 4 (Google). The smartphone operating systems (iOS 13 and Android 10) and VA software (Google Assistant [app version 03/09/2018 update], Alexa [app version 01/14/2020 update], Cortana [app version 11/29/2019 update]) were up to date at the time of this study (February 2020). The language of the phones and apps was set to US English. We used factory reset devices with a new research account, which had no search history, to minimize any bias that may occur from personalization.

We queried 14 frequently asked questions (FAQ) about PPD curated by ACOG [16], which also provides patient-focused answers for each question. The questions are listed in Table 1. One coauthor, JL (female), who is US born, recorded all questions using a 2015 MacBook Air (Apple Corp) MacOS Mojave with Garage Band 10.3.5 using a Yeti Blackout microphone model A00121 (Baltic Latvian Universal Electronics LLC). SY (male) tested 2 questions and confirmed that there was no difference in response from the VAs between the voices of JL and SY. Since the focus of the study is on the clinical advice given by a VA, JL’s voice was used for all recordings. We played back the recordings using the internal speakers in the MacBook Air for each of the 4 VAs to minimize any bias related to the prompt, following the methods in Palanica et al [17]. Recordings and play back occurred in a private office. Each response from the VA was recorded and evaluated in 3 categories: accurate recognition of the query (yes/no), presence of a verbal response (yes/no), and clinically appropriate advice provided (yes/no).

**Table 1:** Occurrence of clinically appropriate advice provided by voice assistants for postpartum depression questions.

Number	Question	Clinically appropriate advice			
		Apple Siri	Amazon Alexa	Google Assistant	Microsoft Cortana
1	Can antidepressants be passed to my baby through my breast milk?	–^a^	–	**+** ^b^	–
2	Can antidepressants cause side effects?	–	–	–	–
3	How is postpartum depression treated?	–	**+**	–	**+**
4	How long do the baby blues usually last?	–	**+**	–	**+**
5	If I think I have postpartum depression, when should I see my health care professional?	–	–	–	–
6	What are antidepressants?	**+**	–	–	–
7	What are the baby blues?	–	–	**+**	–
8	What are the types of talk therapy?	–	–	–	–
9	What can be done to help prevent postpartum depression in women with a history of depression?	–	–	–	–
10	What causes postpartum depression?	–	**+**	–	–
11	What happens in talk therapy?	–	**+**	–	–
12	What is postpartum depression?	**+**	–	**+**	–
13	What support is available to help me cope with postpartum depression?	–	–	–	–
14	When does postpartum depression occur?	–	–	–	**+**

^a^–: no clinically appropriate advice.

^b^+: clinically appropriate advice.

Accurate recognition is the ability of a VA to correctly transcribe the spoken query. Verbal response is the narration and summary of processed web information by the VA. We determined clinically appropriate advice by comparing clinical communication themes found in the VA response and the ACOG FAQ answer. Clinical communication between a provider and patient is the foundation for delivering quality care and is patient-centered, uses plain language without overly complex terms, and, if applicable, provides anticipatory guidance on when to seek medical help [18]. Two board certified physicians (SY, JL) compared the presence of these 3 themes in the ACOG FAQ answer to the VA response. If all the themes that appeared in the ACOG FAQ answer appeared in the VA response, the VA response was marked as clinically appropriate. JL is board certified in Pediatrics and Pediatric Gastroenterology. SY is board certified in Pediatrics and Internal Medicine. Both SY and JL routinely screen for mental health disorders and have over 15 years cumulative experience in answering patient-focused clinical questions. Disagreements were resolved through discussion. We calculated the Cohen kappa interrater reliability using SPSS Statistics version 26 (IBM Corp) [19]. We quantified the responses in each category by aggregating the frequency of positive results in percentages. We compared the category proportions using the Fisher exact test. To control for the false discovery rate in multiple comparisons, we selected a *P* value with an adjusted alpha of .05 or less to determine statistical significance [20].

## Results

### Responses to Postpartum Depression Queries

#### Accurate Recognition

**Figure 1:**
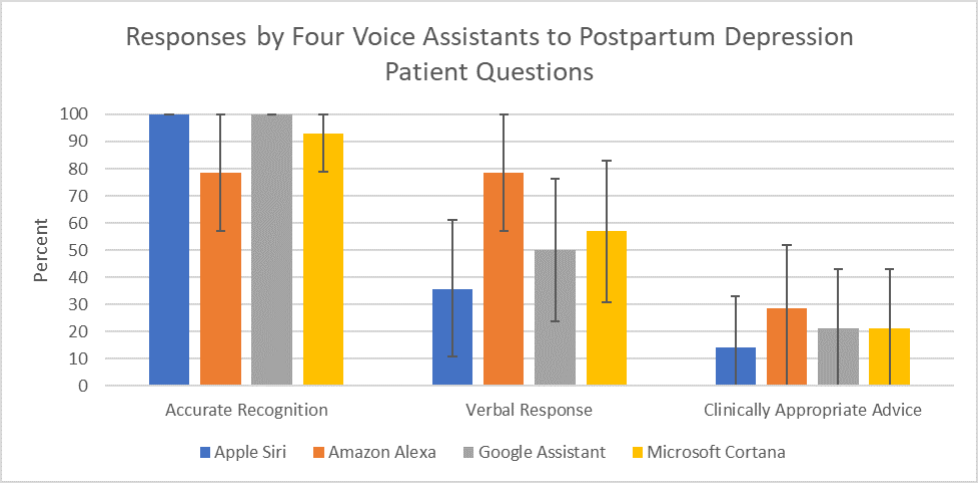
Responses from four principal voice assistants (Apple Siri, Amazon Alexa, Google Assistant, and Microsoft Cortana) to questions regarding postpartum depression as categorized by accurate speech recognition, the presence of a verbal response, and whether the advice was clinically appropriate.

All four VAs performed well when recognizing the queries (Figure 1). Specifically, when prompted, Apple Siri and Google Assistant displayed 100% accurate recognition, whereas Microsoft Cortana displayed 93% (13/14; 95% CI 79.4-100) accuracy and Amazon Alexa 79% (11/14; 95% CI 57.1-100). The accurate recognition differences were not statistically significant (Table 2).

**Table 2:** Fisher exact test comparing voice assistant pairs for accurate speech recognition, the presence of a verbal response, and whether the advice was clinically appropriate.

Voice assistant comparison		Acc rec^a^, n (%)	*P* value	Verb resp^b^, n (%)	*P* value^c^	Clin app adv^d^, n (%)	*P* value
**Comparison 1**		—	.22	—	.05	—	.65
	Apple Siri	14 (100)	—	5 (36)	—	2 (14)	—
	Amazon Alexa	11 (79)	—	11 (79)	—	4 (29)	—
**Comparison 2**		—	>.99	—	.71	—	>.99
	Apple Siri	14 (100)	—	5 (36)	—	2 (14)	—
	Google Assistant	14 (100)	—	7 (50)	—	3 (21)	—
**Comparison 3**		—	>.99	—	.45	—	>.99
	Apple Siri	14 (100)	—	5 (36)	—	2 (14)	—
	Microsoft Cortana	13 (93)	—	8 (58)	—	3 (21)	—
**Comparison 4**		—	.22	—	.24	—	>.99
	Amazon Alexa	11 (79)	—	11 (79)	—	4 (29)	—
	Google Assistant	14 (100)	—	7 (50)	—	3 (21)	—
**Comparison 5**		—	>.99	—	.42	—	>.99
	Amazon Alexa	11 (79)	—	11 (79)	—	4 (29)	—
	Microsoft Cortana	13 (93)	—	8 (58)	—	3 (21)	—
**Comparison 6**		—	>.99	—	>.99	—	>.99
	Google Assistant	14 (100)	—	7 (50)	—	3 (21)	—
	Microsoft Cortana	13 (93)	—	8 (58)	—	3 (21)	—

^a^Acc rec: accurate recognition.

^b^Verb resp: verbal response.

^c^After false discovery rate correction, no comparison was statistically significant.

^d^Clin app adv: clinically appropriate advice.

#### Verbal Response

For each query, verbal response by the VAs ranged from at least 36% (5/14; 95% CI 10.6-60.8) of the questions (by Siri) to 79% (11/14; 95% CI 57.1-100) of the questions (by Alexa). The difference between VAs for the presence of a verbal response was not statistically significant (Table 1). If no verbal response was given, the transcribed query was either treated as a text-based web search providing a list of hyperlinks for the user to follow or the VA stated that it did not know the answer. The following proportions of unanswered queries were treated as a web search: 8/9 for Siri, 7/8 for Google Assistant, and 2/6 for Cortana. Alexa provided a verbal response to each recognized query and therefore had no unanswered queries.

#### Clinically Appropriate Advice

Clinically appropriate advice was provided 14% (2/14; 95% CI 0.0-32.6) of the time by Siri, 29% (4/14; 95% CI 5.9-52.2) by Alexa, and 21% (3/14; 95% CI 0.0-42.9) by Google Assistant and Cortana (Figure 1). There was no statistical difference between devices (Table 2). Taken together, the VAs provided clinically appropriate advice for 64% (9/14; 95% CI 39.1-89.4) of the questions (Table 2). The interrater reliability score (Cohen kappa) among the raters was .87 (*P*<.001; 95% CI 0.69-1.05).

### Additional Voice Assistant Observations

**Figure 2:**
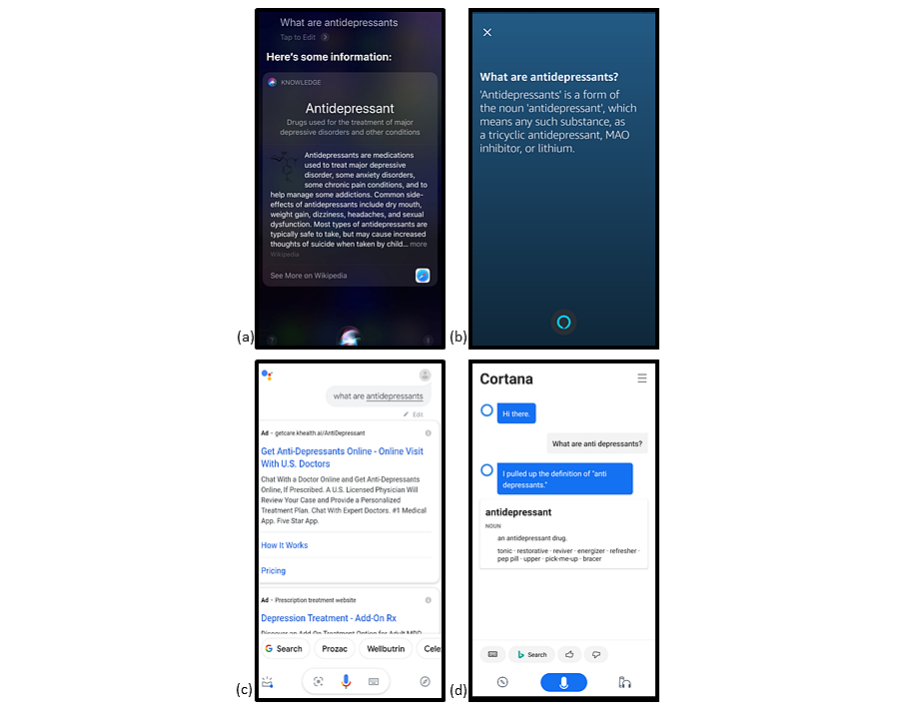
Screenshots from all four voice assistants showing responses to the query “What are antidepressants?” (a) Apple Siri, (b) Amazon Alexa, (c) Google Assistant, and (d) Microsoft Cortana.

We selected one query for which all VAs provided a different response: “What is an antidepressant?” When advice was provided, Siri narrated a short paragraph that was viewable on the screen with a hyperlink to more information (Figure 2a). Alexa displayed a brief explanation without a web link (Figure 2b). Google Assistant displayed a list of web links with advertisements at the top (Figure 2c). Cortana provided an accurate but overly complicated description of the chemical properties of antidepressants as compared with a clinical visit discussion (Figure 2d).

Notably, Google Assistant displayed advertisements for just under half (5/14, 36%) of the queries. Frequently the advertisements from Google Assistant occupied the center of the screen, pushing the reported advice or web search to the bottom (Figure 2c). In one instance, Google Assistant provided an advertisement for a luxury mental health treatment center located in Malibu, California for the query, “How is postpartum depression treated?” Microsoft Cortana was the only other VA to offer advertisements. For some questions such as “What happens in talk therapy?” and “What are the baby blues?”, Alexa and Siri responded with pop culture advice and references, which were not relevant to mental health. The list of VA responses is available in Multimedia Appendix 1.

## Discussion

### Principal Findings

All four VAs performed well at correctly recognizing common questions related to PPD, but no VA achieved even a 30% threshold for providing clinically appropriate PPD information. Specifically, accurate recognition of a PPD question ranged from 79% to 100% among all VAs. A verbal response to a PPD question was given between 36% and 79% of the time, while clinically appropriate advice was only given between 14% and 29% of the time. No single VA outperformed any other VA across performance categories.

The sharp dropoff from accurate speech recognition of a query to clinically appropriate advice indicates that while voice recognition of medical queries continues to improve [17,21], improvement in appropriate clinical advice to end users is essential. When considering the four VAs together (Table 1), that number increases to 64% (9/14). This finding suggests that if these services were to integrate their methods and work collaboratively, their performance could double or triple. For example, Apple and Google collaborated on contact tracing in response to the COVID-19 pandemic [22] demonstrating a willingness by technology companies to combine efforts on essential health issues.

While VAs are not approved medical devices, consumers are nonetheless using VAs to answer medical questions, and they should therefore provide accurate medical advice [5,23,24]. In some cases, the VA referenced websites of health care institutions or government sites such as Mayo Clinic and US Department of Health and Human Services, but in other cases the VA provided references from other websites, such as Wikipedia. These examples illustrate how VAs require improvement to be more context-aware in responding to questions. Especially in the medical domain, the VA interface design could be improved following a similar training protocol to medical students. Medical students are taught to follow-up patient questions with open-ended statements, such as, “Tell me more about that,” which often provides more information than direct queries [25]. This follow-up questioning is an essential step toward improving VA responses in mental health disorders, including PPD.

Advertisements offered by VAs, while also troubling, simply indicate that a reasonable pay structure should be developed where technology companies can recoup their development costs. However, if advertisements continue, a reporting and control mechanism should be in place to identify bias toward a specific treatment or service. Most academic centers have strict policies on accepting free material from vendors to maintain an unbiased stance [26-28].

### Limitations

Our study was limited by our use of grammatically correct, well-structured, well-understood PPD questions. In a real-world application, these exact questions may not represent the variation in language, accent, and education of PPD patients. Hence, the results represent the best-case scenario for VAs when used for PPD. Second, physician assessors subjectively interpreted the ACOG FAQ and decided clinically appropriate advice of the VA using globally accepted components of clinical communication. An alternative study could apply a more stringent rubric to the comparison. Third, we evaluated each question and response as a single unit and did not challenge the VAs with a conversation. An alternative study would be to ask a series of questions to build on the previous question and response and eventually to evaluate the advice as a whole. While the interaction is an important component of VA technology, this study can be seen as a test of the VA knowledge base as it pertains to PPD. Fourth, VA medical advice should adhere to local clinical advice. For example, while we used the ACOG FAQ in this study, the responses to the questions may not be appropriate for users in Africa or Asia [29]. Fifth, our study relied on a binary metric of clinically appropriate advice for each VA response. However, a deeper look at each VA response should include an assessment on whether the response provided or omitted information that was potentially dangerous to patients. Finally, sample sizes are small, so *P* values may not effectively capture differences in VA performance. For example, when comparing Apple Siri and Amazon Alexa for the presence of a verbal response, the calculated power was .49, indicating a low probability of detecting a difference. While a larger sample size is preferred, the goal of this project is to compare the 14-question ACOG FAQ against advice from VAs.

### Future Suggestions

Even though the performance of VAs in responding to PPD currently falls short, VA-based interventions for PPD present a unique opportunity in the long term, as also supported by Kocaballi et al [4] and Miner et al [7]. VAs could support mothers with PPD by addressing misperceptions about the disorder, performing an initial screening, and discussing possible treatment in the privacy of their homes [12]. In light of our findings, we suggest three ways to improve VA performance in mental health disorders and PPD.

First, the lack of clinically appropriate advice showed that clinical organizations should partner with technology companies to develop content and design the user experience. Clinical organizations already produce many patient-facing informational documents found through web searches and VA skills [30]. These organizations should take further steps to develop evidence-based content specifically for VAs, realizing that these devices are not only popular but can also provide meaningful interactions with patients. For example, Siri provided a definition of “baby blues” from pop culture rather than following up with another question to understand the context better.

Second, training VA artificial intelligence with personal information can improve the ability to screen patients and provide context-aware and personalized responses. While this may present privacy challenges, previous successful artificial intelligence implementations in medicine [31] and current VA apps compliant with personal health information protection laws (ie, Health Insurance Portability and Accountability Act) [32] prove that leveraging VA artificial intelligence shows promise in delivering personalized care. For PPD, this effort can lead to better identification of risk factors. Because the US Preventive Services Task Force recommends referral for treatment of PPD after identification of a single risk factor, VA-supported identification could assist providers by marking which of their patients are at risk for PPD. In a future case for PPD, one can imagine a VA moderating screening, counseling a patient on possible treatment options, and assisting providers in decision making with the knowledge gathered through screenings.

Last, public health organizations should partner with technology companies to encourage the delivery of medical care through VAs [33]. In the recent COVID-19 pandemic, the Centers for Disease Control and Prevention used Microsoft’s Healthcare Bot service (text-based) to deliver a relevant and up-to-date information and self-assessment mechanism to the public [34]. This situation is a first-rate example of public-private collaboration in delivering necessary health content through conversational agents. Similar strategies should be established in long-term collaboration in curating and providing PPD and mental health support through VA. To enable successful collaboration, legal and regulatory actions should be developed for digital care delivery, which improved telemedicine during COVID-19 [35].

### Conclusion

VAs accurately recognize speech but cannot give clinically appropriate advice for PPD. PPD is a mental health disorder that presents an opportunity for digital health intervention through VAs. By increasing the conversational abilities of VAs, partnering with health organizations to improve the content of these agents, and integrating these agents with the electronic health record, VAs may be a valuable tool to address patients’ misperceptions, screen patients for depression, and initiate a prompt referral to qualified health providers.

## Appendix

Multimedia Appendix 1 Voice assistant (Apple Siri, Amazon Alexa, Google Assistant, Microsoft Cortana) responses to postpartum depression questions.

## Abbreviations

ACOG: American College of Obstetricians and Gynecologists
FAQ: frequently asked questions
PPD: postpartum depression
VA: voice assistant
